# Aberrant DNA methylation in multiple myeloma: A major obstacle or an opportunity?

**DOI:** 10.3389/fonc.2022.979569

**Published:** 2022-08-18

**Authors:** Catharina Muylaert, Lien Ann Van Hemelrijck, Anke Maes, Kim De Veirman, Eline Menu, Karin Vanderkerken, Elke De Bruyne

**Affiliations:** Department of Hematology and Immunology-Myeloma Center Brussels, Vrije Universiteit Brussel, Brussels, Belgium

**Keywords:** multiple myeloma, epigenetics, DNA methylation modifiers, MM cell plasticity, DNMTi

## Abstract

Drug resistance (DR) of cancer cells leading to relapse is a huge problem nowadays to achieve long-lasting cures for cancer patients. This also holds true for the incurable hematological malignancy multiple myeloma (MM), which is characterized by the accumulation of malignant plasma cells in the bone marrow (BM). Although new treatment approaches combining immunomodulatory drugs, corticosteroids, proteasome inhibitors, alkylating agents, and monoclonal antibodies have significantly improved median life expectancy, MM remains incurable due to the development of DR, with the underlying mechanisms remaining largely ill-defined. It is well-known that MM is a heterogeneous disease, encompassing both genetic and epigenetic aberrations. In normal circumstances, epigenetic modifications, including DNA methylation and posttranslational histone modifications, play an important role in proper chromatin structure and transcriptional regulation. However, in MM, numerous epigenetic defects or so-called ‘epimutations’ have been observed and this especially at the level of DNA methylation. These include genome-wide DNA hypomethylation, locus specific hypermethylation and somatic mutations, copy number variations and/or deregulated expression patterns in DNA methylation modifiers and regulators. The aberrant DNA methylation patterns lead to reduced gene expression of tumor suppressor genes, genomic instability, DR, disease progression, and high-risk disease. In addition, the frequency of somatic mutations in the DNA methylation modifiers seems increased in relapsed patients, again suggesting a role in DR and relapse. In this review, we discuss the recent advances in understanding the involvement of aberrant DNA methylation patterns and/or DNA methylation modifiers in MM development, progression, and relapse. In addition, we discuss their involvement in MM cell plasticity, driving myeloma cells to a cancer stem cell state characterized by a more immature and drug-resistant phenotype. Finally, we briefly touch upon the potential of DNA methyltransferase inhibitors to prevent relapse after treatment with the current standard of care agents and/or new, promising (immuno) therapies.

## Introduction

Multiple myeloma (MM) is an incurable B-cell malignancy characterized by the uncontrolled proliferation and accumulation of malignant plasma cells (PC) in the bone marrow (BM) (1). MM accounts for 1% of all cancers and around 10% of all hematological cancers, being the second most common hematological malignancy (2). In 2020, 176,404 new cases of MM and 117,077 deaths caused by MM (accounting for 1.2% of all cancer deaths) were observed worldwide (3). Age is the most significant risk factor, as MM occurs most frequently in the elderly population with the median age of diagnosis being 65. Consequently, due to an aging population in western countries, the global burden of MM is expected to further increase in the following years (4).

MM is often preceded by a premalignant condition called MGUS or monoclonal gammopathy of unknown significance. An abnormal increase of one type (clone) of PCs can be observed in individuals presenting with MGUS, resulting in the excessive production of one specific immunoglobulin (monoclonal antibody/mAb) termed the M-protein or M-component (5). MGUS progresses to MM at a rate of 1% a year (6, 7). A minor group of the elderly population also suffers from smoldering multiple myeloma (SMM), which is an intermediate phenotype between MGUS and MM. SMM has a 10% risk of developing into MM within the first five years, 5% per year for the following five years, and 1% per year after ten years (8). Patients evolving from MGUS and SMM to MM start to exhibit specific symptoms. In general, MM is diagnosed based on the presence of ≥10% clonal BM plasma cells (BMPC) and the presence of at least one of the MM defining events (MDE). MDE include a serum free light chain (FLC) ratio of ≥ 100, a proportion of clonal BMPC of ≥60%, the detection of one or more focal lesions through MRI, and the presence of one or more CRAB symptoms, including hypercalcemia, renal failure, anemia, and lytic bone lesions (9, 10).

Although MM remains an incurable disease, new treatments developed over the last years have significantly increased the median life expectancy by six to ten years. There are two major lines of treatment options based on transplantation eligibility (age and comorbidities) and risk stratification (11). For patients that are fit enough, first line treatment consists of an induction therapy combining two or three standard of care (SoC) agents, including immunomodulatory drugs (IMiDs; lenalidomide), corticosteroids (dexamethasone), proteasome inhibitors (PI; bortezomib; Bz), and/or monoclonal antibodies (daratumumab, elotuzumab) (10). This induction therapy is then followed by a high dose melphalan treatment combined with an autologous stem cell transplantation (SCT) (12). The second group, people that are not eligible for the SCT, will only be treated with different combinations of the previously mentioned drugs. Although most patients initially respond very well to treatment, most of them will eventually relapse and with each new round of therapy they will respond less until they become completely refractory. Therefore, new therapies are still being developed, like chimeric antigen receptor (CAR) T-cell therapy, antibody drug conjugates (ADC), and T-cell engagers, which are currently being tested in clinical trials (13–16). Although clinical trials showed encouraging results, patients are still relapsing. Hence, there is still no definite treatment to cure MM.

One of the most important reasons for relapse is the development of drug resistance (DR) against all SoC agents. DR can be established on different levels. The close interaction between MM cells and the BM niche is one of the major mechanisms playing a role in the development of DR. Two categories of BM-related DR exist, namely cell adhesion mediated DR (CAM-DR), caused through the interactions between the MM cells and the cellular compartment (BM stromal cells, endothelial cells, osteoblasts, osteoclasts, and immune cells) and/or extracellular matrix, and soluble factor mediated DR (SFM-DR), caused through the interactions between the MM cells and the non-cellular compartment (cytokines, growth factors, chemokines, and exosomes) (17, 18). In addition, changes in the MM cells themselves also lead to DR. These changes can be due to multiple causes: genetic and epigenetic abnormalities, disruptions in intracellular signalization pathways, aberrant metabolism, and aberrant drug transport (19–21).

**Figure 1:**
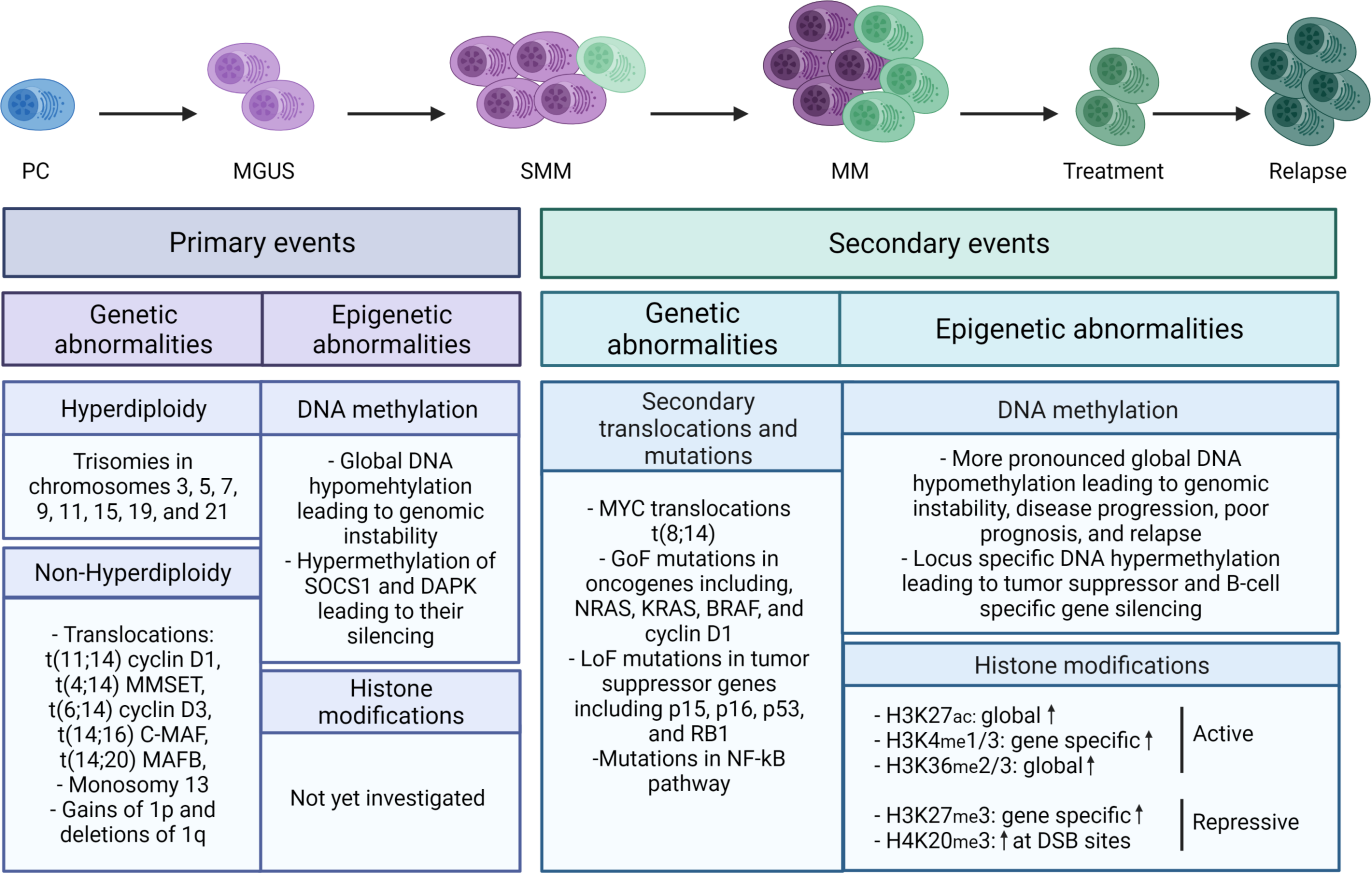
Schematic overview of the genetic and epigenetic abnormalities in MM. Primary (epi)genetic events are driving MM onset, while the secondary events are fostering MM progression and relapse. Primary genetic events include both hyperdiploid and non-hyperdiploid abnormalities, while primary epigenetic events are mainly characterized by global DNA hypomethylation and promotor specific hypermethylation of the tumor suppressors SOCS1 and DAPK. Secondary genetic events include non-recurrent secondary translocations, gain-of-function (GoF) mutations in oncogenes, and loss-of-function (LoF) mutations in tumor suppressor genes, while on the epigenetic level more pronounced global DNA hypomethylation and locus specific hypermethylation are observed together with increased (both global and locus specific) histone mark levels, including H3K27Ac, H3K4me1/3, H3K36me2/3, H3K27me3, and H4K20me3. The blue cell represents a normal plasma cell, while the purple and green cells represent respectively MM cells with primary and secondary events. The color gradation represents the increase in abnormalities in each stage. DSB, double strand breaks.

On a genetic level, a large intra- and interpatient clonal heterogeneity is observed. Patients can roughly be divided into two groups, namely the hyperdiploid group and the non-hyperdiploid group. The hyperdiploid group is characterized by odd-numbered chromosome trisomies involving chromosomes 3, 5, 7, 9, 11, 15, 19, and 21, while the non-hyperdiploid group is characterized by primary translocations involving chromosomes t(11,14)(q13;q32), t(4,14)(p16;q32), t(6,14)(p21;q32), t(14,16)(q32;q23), and t(14,20)(q32;q12) (**Figure 1**) (22, 23). Patients in the hyperdiploid group and patients harboring the t(6,14)(p21;q32) and t(11,14)(q13;q32) translocation have a more favorable prognosis, while patients harboring one of the other primary translocations have a poor prognosis (23). On top, many non-recurrent secondary translocations and mutations are acquired during disease progression, including the translocation of the MYC gene, gain-of-function mutations in several oncogenes (NRAS, KRAS, BRAF, and CCND1), and loss-of-function mutations in tumor suppressor genes such as p15, p16, and P53 (**Figure 1**) (24–28).

Apart from being a genetic disease, MM is also deregulated on an epigenetic level (11). In MM, sequencing and gene expression profiling studies have identified numerous epigenetic defects (also called ‘epimutations’) including defects in DNA methylation and posttranslational histone modifications and aberrant expression of miRNAs, probably resulting from genetic defects and/or deregulated expression of the epigenetic modifiers (writer, reader, and eraser proteins) (**Figure 1**) (29). These epimutations play an important role in disrupting critical regulatory networks in MM, such as the Wnt/β-catenin, JAK/STAT, Cyclin/CDK/Rb, and DAPK/p14ARF/p53 pathway amongst others (30). Hence, these epimutations are well-known to contribute to genomic instability, disease progression, and high-risk disease in MM (11, 31). More recently, increasing evidence has been provided that epiplayers also play an important role in MM cell DR (11, 31). In this review, we discuss recent advances in our understanding of the role of aberrant DNA methylation and DNA methylation modifiers in MM pathogenesis. Furthermore, we describe the role of these modifiers in MM cell DR and disease progression and the potential of combining DNA methylation modifier inhibitors with SoC or novel agents to avoid relapse.

## DNA methylation

### Normal DNA methylation patterns

**Figure 2:**
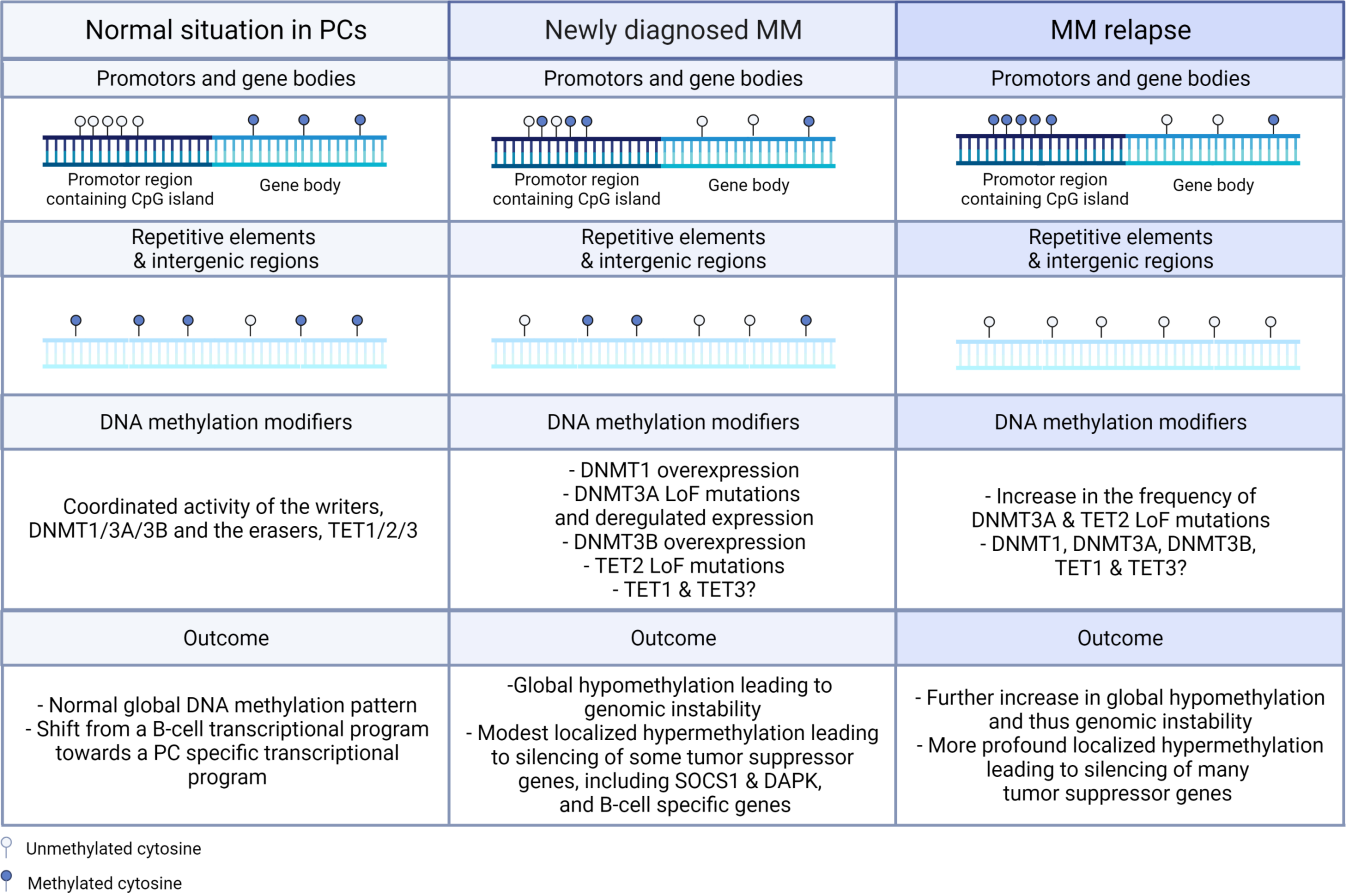
Schematic representation of the changes in global and gene specific DNA methylation patterns and DNA methylation modifiers in newly diagnosed and relapsed MM. In normal circumstances, CpG islands found in the promotor regions are in general not methylated, while the CpG dinucleotide in gene bodies, intergenic regions, and repetitive elements are mostly methylated. In MM, global hypomethylation is found in gene bodies, intergenic regions, and repetitive elements, while hypermethylation is observed in the CpG islands found in the promotor regions of tumor suppressor genes. In the relapsed settings, these events are even more pronounced. On the levels of the DNMT and TET enzymes, overexpression of DNMT1 and DNMT3B and loss-of-function (LoF) mutations in DNMT3A and TET2 are observed in newly diagnosed patients and the frequency of the LoF mutations in DNMT3A and TET2 are even further increased in the relapse setting. In healthy PCs, normal global methylation patterns are observed with a shift from the B-cell transcriptional program towards the PC specific transcriptional program, while in MM cells global hypomethylation leading to genomic instability and localized hypermethylation leading to silencing of tumor suppressor and B-cell specific genes are observed. PC, plasma cell.

DNA methylation, one of the most studied epigenetic modifications so far, is a vital process for mammalian development and plays an essential role in many biological processes, including cellular differentiation and tissue-specific gene expression, X-chromosome inactivation, genomic imprinting, and silencing of transposable elements (30). Methylation of the DNA is a non-random process in which a methyl group from the methyl donor S-adenosyl-L-methionine (SAM) is covalently added on the fifth carbon position of cytosine residues (5mC) that are directly followed by guanine in the 5’ to 3’ direction, the so-called CpG dinucleotide (32). Methylation of the DNA is generally accepted to result in increased nucleosome compactness and thus gene silencing. However, transcriptional activation upon DNA methylation resulting from, in all likelihood, the recruitment of transcription factors (TF) that preferentially bind methylated DNA (such as RFX) has also been observed and this especially upon 3’ methylation in embryonic stem cells (ESC) (33). In healthy cells, CpG dinucleotides are mainly found in intergenic regions, gene bodies, and repetitive elements and 70-80% of these CpG dinucleotides are methylated, leading to transcriptional inactivation of e.g. transposable and viral elements, which is necessary to preserve the normal integrity of the genome (**Figure 2**) (34, 35). In addition, 10% of the CpGs can be found in a large number of consecutive CpG dinucleotides, called CpG islands. These CpG islands are mainly located at the transcription start sites of promotors and it is estimated that 50-60% of the gene promotors contain CpG islands (**Figure 2**) (36). Although these CpG islands are most often unmethylated in healthy cells thus permitting gene expression, methylated CpG islands can also be found and this especially in imprinted genes and in multiple X-chromosome genes that are inactivated in females (37). In contrast, in cancer cells, these normal DNA methylation patterns are often completely disturbed, with a general shift towards global hypomethylation in the intergenic regions and locus specific hypermethylation of the CpG island in the promotor region of tumor suppressor genes. The abnormal DNA methylation patterns in MM will be discussed in more depth in the following chapter (Chapter 3).

### DNA methylation modifiers

The DNA (de)methylation process is mediated by three types of chromatin modifying enzymes, namely the DNA methylation writers, readers, and erasers. The writers, involving the DNA methyltransferase (DNMT) family, are responsible for establishing the 5mC mark. The readers recognize and bind the methylated DNA and influence chromatin compactness and gene expression by recruiting several activator or repressor complexes. Modification and removal of the 5mC marks are established by the erasers, involving mainly the TET enzymes.

#### -Writers

**Figure 3:**
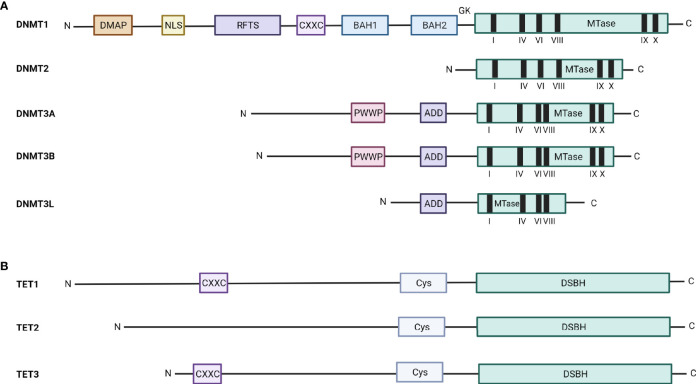
Schematic overview of the structure of the DNMT and TET enzymes. The structure and different domains of **(A)** the five DNMT family members, DNMT1, DNMT2, DNMT3A, DNMT3B, and DNMT3L, and **(B)** the three TET family members, TET1, TET2, and TET3, are depicted. DMAP, DNA methyltransferase 1-associated protein binding domain; NLS, nuclear localization signal; RFTS, replication foci-targeting sequence; CXXC, Zn finger CXXC-domain; BAH, bromo-adjacent homology; GK, glycine-lysine repeat; MTase, methyl transferase; PWWP, proline-tryptophan-tryptophan-proline; ADD, ATRX-DNMT3A/B-DNMT3L; Cys, cysteine-rich domain; DSBH, double-stranded β-helix domain.

The DNA methyltransferase family consist out of 5 DNMTs, encompassing DNMT1, DNMT2, DNMT3A, DNMT3B, and DNMT3L. However, only DNMT1, DNMT3A, and DNMT3B are able to methylate the DNA. DNMT1, DNMT3A, and DNMT3B all have a regulatory N-terminal domain and a catalytic C-terminal domain as illustrated in **Figure 3**, with the N-terminal domain being responsible for their distinct activity. The N-terminal domain of DNMT1 contains several (sub)domains, including a DNA methyltransferase 1-associated protein (DMAP)-binding domain, which binds DMAP and can consequently interact with the histone deacetylase HDAC2; a nuclear localization signal (NLS); a replication foci-targeting sequence (RFTS), which localizes DNMT1 to the DNA replication fork; a Zn finger CXXC-domain, which recognizes unmethylated CpG containing DNA; 2 bromo-adjacent homology (BAH) domains with unknown function; and a glycine-lysine (GK) repeat, which links the N-region to the C-region. In contrast, the N-terminal domain of DNMT3A and DNMT3B both consist out of a proline-tryptophan-tryptophan-proline (PWWP) domain needed for heterochromatin localization and an ATRX-DNMT3A/B-DNMT3L (ADD) domain that is necessary for the interaction of DNMT3A/B with unmethylated H3K4 (histone 3 lysine 4) (38). The C-terminal domain is quite similar for DNMT1, DNMT3A, and DNMT3B and is responsible for the deposition of the methylation mark in the presence of SAM.

The DNMTs are recruited to the DNA in both a gene/locus specific and a non-specific manner, which is regulated through the binding of the DNMTs with many different binding partners, which, in turn, regulate their activity. Specific recruitment is regulated by polycomb (PcG) proteins and TF, but is also observed upon DNA repair (DNMT1 recruitment *via* PCNA), while the unspecific recruitment involves the cooperation with heterochromatin readers or replication associated proteins (39).

In general, DNMT1 is considered a maintenance methyltransferase, meaning that it is responsible for maintaining CpG methylation after DNA replication, as it preferentially recognizes hemimethylated DNA (40). DNMT1 is thus mainly responsible for the maintenance of DNA methylation patterns upon completion of the embryonic cell fate specification and is strongly expressed in almost all adult tissues (41, 42). Knock-out (KO) of Dnmt1 in mice showed that Dnmt1 is necessary for embryonic development, gene imprinting, and X-chromosome inactivation (43). Furthermore, homozygous mutations in Dnmt1 in mice resulted in a delayed development with mice dying before birth, thus (further) showing that DNMT1 is essential for mammalian development and normal cell function (44). In contrast, DNMT3A and DNMT3B are mainly seen as *de novo* DNA methyltransferases, that are able to recognize unmethylated DNA and establish new DNA methylation patterns during embryogenesis (32, 45, 46). More recently, however, DNMT3A and DNMT3B have been suggested to contribute to the maintenance of the DNA methylation patterns in differentiated cells as well, although their catalytic activity appears to be 20x lower than that of DNMT1 (43). Both DNMT3A and DNMT3B are highly expressed in undifferentiated embryonic stem cells and the expression declines after birth. Yet, distinct functions between DNMT3A and DNMT3B are observed. DNMT3B is highly expressed at E7.5 embryonic development, thus playing a critical role early in the development, while DNMT3A is highly expressed at E8.5 and E9.5 playing a role later in the development and even after birth (32, 45, 46). In line, DNMT3B knockdown (KD) mice died before birth, while DNMT3A KD mice died four weeks after birth. Moreover, DNMT3A is important for methylation of imprinted genes, while DNMT3B is important for methylation of centromeric, pericentromeric, and subtelomeric regions (39, 47). This difference in function can be explained by the fact that there is only a 28% homology in the N-terminal domain between DNMT3A and DNMT3B. Importantly, DNMT3A and DNMT3B not only play a role during embryogenesis, but also during the normal life span (48). In adults, DNMT3A is expressed in almost all tissues, while high DNMT3B levels are mainly observed in the testis, thyroid, and the bone marrow (49). Within the bone marrow, DNMT3B expression levels vary among the different cell types, with the CD34+ cells (the hematopoietic stem cells) showing high DNMT3B levels compared to the more differentiated cell types (41).

Importantly, as a result of alternative splicing, multiple isoforms with distinct activities can be found for DNMT1, DNMT3A, and DNMT3B (**Table 1**) (61). In mice, three different Dnmt1 isoforms are reported, namely Dnmt1s, Dnmt1o, and Dnmt1p. These isoforms are the result of alternative splicing of the N-terminal exons, with each of the isoforms having their own specificity. DNMT1s is specific to somatic cells, while Dnmt1o and Dnmt1p are specific to oocytes and pachytene spermatocytes respectively (50). However, these DNMT1 isoforms have not yet been identified in humans. For DNMT3A, two isoforms have been described, namely DNMT3A1 and DNMT3A2. DNMT3A1 is the full-length protein, while DNMT3A2 lacks 223 amino acids at the N-terminal end (51). Although both isoforms have the PWWP, ADD domain, and the identical catalytic domain, a difference in DNA methylation activity is observed; with DNMT3A1 having a higher DNA-binding and DNA methylation activity compared to DNMT3A2 (62). Moreover, DNMT3A1 also shows a higher affinity for heterochromatin, while DNMT3A2 has more affinity for euchromatin (51, 52). For DNMT3B, more than forty different isoforms have been reported and are known to be expressed in a tissue specific manner. However, so far, only nine isoforms have been described in literature in more detail (42, 49, 54–60). The activity of these isoforms is determined by the preservation of the highly conserved catalytic domain. DNMT3B1 and DNMT3B2 are both catalytically active isoforms, while the DNMT3B4 and DNMT3B5 isoforms are catalytically inactive due to a frameshift mutation, leading to an early stop codon causing disruption of the catalytic domain (49, 55). The catalytic inactive isoforms are thought to regulate the activity of the active DNMT3A and DNMT3B isoforms by physical interaction (53). Although alternative splicing is most commonly observed in the C-terminal catalytic region of DNMT3B, it can also be observed in the N-terminal region. DNMT3B isoforms lacking the N-terminal region are called delta (Δ) DNMT3B. At the moment, at least seven different ΔDNMT3B are known (63). As to our knowledge, no difference in activity has been described among these different ΔDNMT3B (64).

**Table 1:** Overview of the DNMT1, DNMT3A, and DNMT3B isoforms.

Splice variant (isoforms)	Tissue specificity of expression (normal, not cancer)	Size difference compared to most typical form: mRNA	Size difference compared to most typical form: protein	Catalytic active	References
**DNMT1**					
Dnmt1s	Somatic cells (Only in mouse model)	Full length	1621 aa	Yes	(50)
Dnmt1o	Oocytes and preimplantation embryos (Only in mouse model)	Smaller than Dnmt1s	Lacking 118 amino acid residues at the N-terminus	Yes	(50)
Dnmt1p	Pachytene spermatocytes (Only in mouse model)	Longer than Dnmt1s	No, since no translation	N.A.	(50)
**DNMT3A**					
DNMT3A1	Ubiquitously expressed in all adult and foetal tissues (low)	Full length	912 aa	Yes	(51, 52)
DNMT3A2	Embryonic stem cells, testis, spleen, and thymus	Lacks first 6 exons	723 aa or 689 aa	Yes	(51, 52)
**DNMT3B**					
DNMT3B1	ESC, embryos, and all tissues except brain, PBMC, and skeletal muscle	Full length	853 aa: canonical sequence	Yes	(42, 49)
DNMT3B2	ESC, embryos, and testis	Lacks exon 10	833 aa	Yes	(49)
DNMT3B3	ESC, embryos, testis, and ubiquitously expressed in normal human tissues	Lacks exon 10, 21, and 22	770 aa: lacks first nine aa of the methyltransferase motif IX	No	(42, 49, 53)
DNMT3B4	All tissues except brain, lung, prostate, and skeletal muscle	Lacks exon 10 and 21	744 aa: lack of methyltransferase motifs IX and X	No	(42, 53, 54)
DNMT3B5	Testis and very low levels in brain and prostate	Lacks exon 10 and 22	812 aa: lack of methyltransferase motifs IX and X	No	(42, 54)
DNMT3B6	Germ cells	Lacks exon 10, 21, and 22	845 aa No lack of methyltransferase motifs	No	(55)
DNMT3B7	Germ cells	Lack of exon 5, 10, 21, and 22	728 aa: No lack of methyltransferase motifs	No, dominant negative	(56–58)
DNMT3B8	Germ cells	Lacks exon 4, 5, 10, 21, and 22	694 aa: No lack of methyltransferase motifs	No	(59)
DNMT3B9	Leukemic specific	Lacks exon 7 and 10	No lack of methyltransferase motifs	Yes, only in cancer	(60)

N.A., not applicable; ESC, Embryonic stem cells; PBMC, peripheral blood mononuclear cell. aa, amino acids.

The two other members of the DNMT family, DNMT2 and DNMT3L, are not able to methylate the DNA. DNMT2 only contains the catalytic C-terminal domain and plays an important role in the methylation of a specific cytosine in tRNA^Asp^. In contrast, DNMT3L lacks both the N-terminal PWWP domain and the catalytic activity of the C-terminal domain and is thus seen as a truncated version of DNMT3B. DNMT3L appears to play an important role as cofactor for DNMT3A/B and is known to increase their methyltransferase activity (65).

#### -Erasers

For a long time, it was believed that DNA methylation resulted in a stable and permanent repression of the genes, but new evidence showed that the DNA methylation marks can be removed making DNA methylation a reversible process. There are two forms of DNA demethylation, namely active and passive demethylation. Passive demethylation is induced upon cell division, when a lower DNMT1 methylation activity is observed. In contrast, active demethylation is initiated by the Ten-eleven translocation (TET) enzymes (66, 67). There are three TET enzymes (TET1-3) with different activity and structure as shown in **Figure 3**. TET1 and TET3 have a N-terminal domain, which contains a CXXC-domain, and a catalytic C-terminal domain, consisting out of cysteine-rich (Cys) and double-stranded β-helix (DSBH) domains. TET2 has the same catalytic C-terminal domain, but lacks the N-terminal CXXC-domain (68). For TET1 and TET3, the N-terminal domain recognizes and recruits them to the methylated DNA, while TET2 recruitment to methylated DNA is facilitated through the interaction with DNA binding proteins, including the tissue-specific TF early B-cell factor 1 (EBF1) (69). The TET enzymes oxidize the 5mC to 5hmC (5-hydroxymethylcytosine), 5fC (5-formylcytosine) and 5caC (5-carboxylcytosine). Final active demethylation is then mediated by the thymine-DNA-glycosylase, which recognizes and excises 5fC and 5caC, followed by base excision repair (BER)-mediated replacement of the modified cytosine by an unmodified one. While all three TET enzymes have similar activity, TET1 and TET2 play a role in the demethylation at specific loci in the primordial germ cells (PGCs), whereas TET3 plays an important role in erasing paternal methylation marks in the male pronuclei of zygotes (38).

Isoforms of the TET enzymes, resulting from alternative splicing, have also been observed; with each isoform having a distinct expression pattern and function. TET1 isoforms encompass TET1 Fl (full length) and TET1s (short) isoform, with TET1s lacking the N-terminal side containing the CXXC-domain. TET1 Fl is observed in early embryos, ESCs, and PGCs, while TET1s is observed in somatic cells. TET2 has two different isoforms, namely TET2 Fl and TET2n, an N-terminus isoform which lacks the C-terminus and thus shows no enzymatic activity. For TET3, three different isoforms exist, namely TET3 Fl, TET3s, and TET3o (ovarian), with TET3s and TET3o both lacking the CXXC-domain. TET3 Fl is specific for neuronal differentiation, while TET3s is highly expressed in neurons and the retina and TET3o is expressed in oocytes and zygotes (70, 71).

#### - Readers

The methylated cytosines will be recognized and bound by the methyl binding proteins. There are three families of methylated DNA binding proteins, namely the methyl-CpG-binding domain (MBD) family, the SET and RING finger associated (SRA) domain protein family, and the methyl CpG binding Zinc finger family. Members of the MBD family include, MeCP2 and MBD1-4. MeCP2, MBD1, MBD2, and MBD4 all contain a methyl-CpG-binding domain (MBD), allowing to recognize and bind methylated DNA. In addition, MBD4 also contains a glycosylase domain, which appears to have a function in DNA mismatch repair. In contrast, MBD3 contains a mutated MBD domain, making it unable to bind to methylated DNA on its own. Instead, MBD3 localization to the DNA is possible since MBD3 is a subunit of the Nucleosome Remodeling Deacetylase (NuRD) complex, which also contains the histone deacetylases 1 and/or 2 (HDAC1/2) amongst others. MeCP2 and MBD1-2 also contain a transcription repressing domain (TRD), which allows the interaction with different repressor complexes and chromatin modifying enzymes, such as HDAC1/2 or the histone methyltransferases (HMT) SUV39h1/HP1 and SETDB1, resulting in further transcriptional repression (38, 72–74) and thus a self-reinforcing loop of silencing (75). The SRA domain proteins include the ubiquitin-like with PHD and Ring Finger Domains (UHRF) 1 and 2. UHRF1 plays a role in the localization of DNMT1 to DNA replication foci and also recognizes histone modifications such as H3K9me3, while UHRF2 preferentially binds to 5hmC (76, 77). The methyl CpG binding Zinc finger family includes ZBTB33, ZBTB38, ZBTB4, Zfp57, Klf4 (Krüppel-like factor 4), WT1 (Wilms tumor protein 1), Egr1 (growth response protein 1), and CTCF (CCCTC-binding factor), which are reviewed in depth by Hudson et al. (78).

## Aberrant DNA methylation in MM

### Abnormal methylation patterns in MM cells

In MM cells, just like cancer cells in general, a disturbed DNA methylation landscape is observed with global hypomethylation leading to genomic instability and localized hypermethylation contributing to silencing of tumor suppressor and B-cell specific genes (**Figure 2**). In general, the changes in DNA methylation patterns are disease-stage specific, with global hypomethylation occurring already in the early stages of the disease, whereas locus specific hypermethylation is more frequent in the more advanced disease stages (79, 80). In line, Walker et al. showed that the determination of the methylation pattern is capable of distinguishing the premalignant (MGUS) from the malignant (MM) conditions (80).

Global DNA hypomethylation is already present in MGUS and newly diagnosed (ND) MM patients and correlates with disease progression and poor prognosis (**Table 2**) (81, 82). In 2009, Bollati et al. found that global hypomethylation of the repetitive elements LINE1, Alu, and SAT alpha in MM is linked with chromosomal instability, with lower methylation levels of LINE1 and SAT alpha being observed in the non-hyperdiploid group compared to the hyperdiploid group. Furthermore, lower levels of methylated Alu and SAT alpha were observed in the t(4,14) group, a group with a worse prognosis compared to the other myeloma cytogenetic subgroups (81). Walker et al. confirmed these findings by showing that the t(4,14) translocation is associated with the strongest heterogeneity in DNA methylation profiles, characterized by the highest amount of hypomethylated regions on the one side and the highest amount of hypermethylated genes on the other side (80). Moreover, they also showed a further decrease in global methylation levels upon disease progression (80). More recently, genome-wide hypomethylation was also observed in patients from the MMRF CoMMpass study, which represents the largest molecular profiling initiative in MM patients, with median global CpG methylation levels of 41% compared to normal PC and B-cells with respectively 71% and 89% of global CpG methylation levels (90).

**Table 2:** Potential biomarkers in MM based on MM signatures and DNA methylation modifiers.

Potential MM biomarkers		
Type	Prognostic value	Reference
**Signatures**		
Global DNA hypomethylation	Poor prognosis	(81, 82)
Epiallele shifts	Poor OS when acquired at time of diagnosis	(83)
Gene-expression based score to predict patient outcome and MM sensitivity towards HDACi and/or DNMTi combination treatment	High DM, HA and combo score linked with worse OS	(84–86)
**DNA methylation modifiers**		
Mutations in any DNA methylation modifier (TET1/2/3, IDH1/2, DNMT1/3A/3B)	Shorter OS	(87)
DNMT3A downregulation	Poor OS	(79)
DNMT3A upregulation	Potential biomarker for tumor progression	(88)
TET2 overexpression	Better OS	(89)

OS, overall survival; DM, DNA methylation; HA, histone acetylation; DNMT, DNA methyltransferase; TET, ten-eleven translocation; IDH, isocitrate dehydrogenase. HDACi, histone deacetylase inhibitor.

In contrast, DNA hypermethylation is mainly observed in the later stages of MM and this mainly in the CpG islands present in the promotor regions of tumor suppressor genes [including RASSF4, p15, p16, p73, TP53, SOCS1, DAPK, SFRP1, SFRP2, VHL, and EGLN3 (**Table 3**)] and enhancer regions of B-cell specific genes (including BCL11A, BATF, EBF1, and PAX5), causing inactivation of these genes (93–118). Importantly, the promotor specific hypermethylation appears to further increase from the ND stage to the relapse stage, with the highest promotor methylation levels being observed in the plasma cell leukemia (PCL) stage. Hypermethylation of SOCS1 and DAPK promotor regions has been identified as early events in the MM pathogenesis, since hypermethylation of these promotor regions has already been observed in the MGUS stage (**Figure 2**). In contrast, hypermethylation of p16, SHP1, and E-CAD are only first observed in the MM stage, thus contributing to disease progression (94, 102, 118). Furthermore, when patients from an early disease stage developed to a later disease stage, the promotor region of seventy-seven (tumor suppressor) genes was found to become hypermethylated, with the hypermethylation of some of these genes, including p16, DAPK, BCL2/BNIP3, and CDH1 (E-CAD) (**Table 3**) correlating with a shorter overall survival (OS) (79, 80). Apart from gene specific hypermethylation, gene specific hypomethylation has also been observed in the oncogenes JAG2 and ABC transporter, thus leading to their aberrant expression (**Table 3**) (91, 92). Furthermore, although it was long assumed that aberrant hypermethylation patterns are primarily present in promotor regions of genes, increasing evidence is now showing that these patterns are also present in gene bodies (Notch1 & GATA3) and intergenic regions (32). In line, Agirre et al. reported profound hypermethylation outside the CpG rich promotors, in the intronic enhancers overlapping with binding sites of B-cell specific TF such as BCL11A, BATF, EBF1, and PAX5 (119).

**Table 3:** List of genes that are hypo- or hypermethylated in MM.

Hypomethylated oncogenes		
Gene	Function	Reference
JAG2	Positive regulator of Notch signaling pathway	(91)
ABC transporter	Role in drug efflux	(92)
Hypermethylated tumor suppressor genes		
Gene	Function	Reference
**p15, p16, p73, TP53**	Cell cycle control	(93–99)
**SFRP1, SFRP2, SFRP4, SFRP5, DKK1, DKK3, APC, WIF1**	Negative regulators of Wnt/B-catenin pathway	(100)
**SOCS1, SHP1**	Negative regulators of IL-6 and JAK/STAT pathway	(93, 94, 101–103)
**DAPK, BCL2/BNIP3, BCL7c, GADD45, XAF1, RB1**	Apoptosis	(93–95, 97, 104–106)
**CDH1 (E-CAD), GJA1, AKAP12, DCC, TIMP3**	Cell adhesion	(93, 94, 97, 104, 107)
**MGMT, hMLH1**	DNA repair	(94, 97)
**RASSF4**	Negative regulator of RAS pathway	(108)
**VHL, EGLN3**	Degradation of the hypoxia-inducible factor–1α (HIF-1α)	(109, 110)
**IRF8**	Transcription factor of the interferon (IFN) regulatory factor (IRF) family	(111)
**GPX3**	Suppresses growth through ROS stabilisation	(112)
**RBP1, RARbeta**	Positive regulator of retinoic acid signaling	(112)
**SPARC**	Role in treatment response; increases sensitivity toward chemotherapy	(112)
**TGFBI**	Role in treatment response; increases sensitivity toward chemotherapy	(112)
**DLC-1**	Negative regulator of the p family of small GTPases	(113)
**TGFbR2**	Positive regulator of TGFβ signaling pathway (TGFβ anti-cancer effects)	(107)
**CD9**	Negative regulator of cancer cell motility and metastasis	(114)
**RASD1**	Role in treatment response; increases sensitivity toward dexamethasone	(115)

Hypermethylation of the tumor suppressor genes marked in purple is correlated with a poor prognosis.

More recently, with the availability of enhanced reduced representation bisulfite sequencing (eRRBS), it has become apparent that the MM epigenome is not only characterized by global hypomethylation and focal hypermethylation of CpG islands, but also by a high degree of intratumoral epigenetic methylation heterogeneity (83). Moreover, when comparing the epiallele composition changes (= epiallele shifts) between normal PCs and MM patients at diagnosis, the extent of these epiallele shifts was found to be highly variable between patients and appeared associated with poor OS when acquired at the time of diagnosis (and this independently of high-risk genetic lesions) (**Table 2**). In addition, comparison of the epiallele shift between matched newly diagnosed and relapsed patients revealed that upon relapse, 42% of the patients showed substantial accumulation of stochastic methylation. Importantly, these stochastic methylation gains were mainly found in bivalent promotors of developmental genes, which are in normal circumstances tightly regulated by the balance between the transcriptionally repressive histone mark H3K27me3 and the active histone mark H3K4me3 and generally free from DNA methylation. This allows flexible regulation of the expression of these developmental genes. However, in B-cell tumors, epigenetic switching from H3K27me3 to DNA methylation is observed at some loci. This switch is also referred to as “Polycomb repression-associated DNA methylator phenotype” or PRAMP. PRAMP decreases the flexibility between the repressive and active state, leading to a more permanent “silenced” state of key regulatory genes (120). Together, these findings suggest that the enhanced stochastic methylation variation makes it possible for MM cells to adapt to their environment (including treatment pressure) to survive (83).

### DNA methylation modifiers in MM cells

The exact reason for the disturbed DNA methylation landscape in cancer cells is currently still unknown, but the most likely explanation is the aberrant expression and/or activity of the DNA methylation modifiers (31, 93). In this review, we will focus on the role of the writers and erasers in MM disease. Overexpression of DNMT1, DNMT3A, and DNMT3B has been observed in many solid and hematological cancers, including MM (121, 122). Moreover, mutations in the DNA methylation modifiers are also observed in MM, correlating with a shorter OS when grouped all together (**Table 2**). Although mutations in DNA methylation modifiers are only observed in 4% of the patients at diagnosis, their frequency appears significantly increased in the relapsed setting and this especially for DNMT3A and TET2 mutations (87). In contrast, mutations in DNMT1/3B and TET1/3 are less frequent in MM.

#### - Writers:

##### • DNMT1

Increased expression of DNMT1 is often found in several solid and hematological cancers and has been linked with a poor prognosis, especially in solid cancers (32). DNMT1 is crucial for faithfully maintaining methylation patterns in human cancer cells and DNMT1 KO leads to severe mitotic defects and even cell death (40). In MM, DNMT1 is also found overexpressed and the levels further increase as the disease progresses, indicating a role for DNMT1 in disease progression (81). In line, KD of DNMT1 using siRNA was shown to decrease MM cell proliferation, due to a G1-phase block and re-expression of SOCS1 and p16, and to increase apoptosis due to cleavage of caspase 3 and PARP (123, 124). A recent study in MM also showed that DNMT1 is responsible for the hypermethylation of tight junction protein 1 (TJP1), a negative regulator of the epithelial-to-mesenchymal transition (EMT), leading to decreased TJP1 levels and poor OS (125). The exact mechanisms behind the aberrant expression of DNMT1 are currently unknown, but studies in other cancers are indicating that the DNMT1 levels could be regulated by miR-148a (126). In addition, some proteins such as the fatty acid-binding protein 4 (FABP4) and nucleolin were also found to regulate DNMT1 levels in hematological cancers (127–130). Inhibition of these proteins resulted in the downregulation of DNMT1 and a decreased clonogenic potential of cancers cells in acute myeloid leukemia (AML) and chronic myeloid leukemia (CML) (128–130).

##### • DNMT3A

Mutations of DNMT3A are frequently observed in different hematological malignancies, including AML (both adult and pediatric), T-cell acute lymphoblastic leukemia (T-ALL), myelodysplastic syndrome (MDS), and T-cell lymphomas (TCL) (131). In AML, DNA sequencing showed that 22.1% of the newly diagnosed patients have mutations in DNMT3A. The most common mutations found in AML patients are missense mutations, especially those observed at the amino acid R882 located in the methyltransferase domain, resulting in a dominant negative effect. Other mutations such as deletions, frameshift, nonsense, and splice-site mutations are also described. Most of these mutations are consistently associated with loss-of-function, leading to genome-wide hypomethylation and correlated with a shorter OS (132). Mutations at R882 are also observed in the DNMT3A isoforms. Overexpression of mutated DNMT3A1 or DNMT3A2V (a DNMT3A2 variant lacking 68 base pairs) in AML cells resulted in increased proliferation rates, while overexpression of normal DNMT3A1 or DNMT3A2 significantly reduced cell proliferation, thus strengthening the hypothesis that DNMT3A has a tumor suppressive role (133). In line with this tumor suppressive role of DNMT3A, a recent study also reported upregulation of DNMT3A in AML patients, correlating with a better leukemia-free and OS (134). Currently, there are only few studies that have focused on DNMT3A in MM. Mutations in the DNMT3A gene are less frequently observed in MM compared to the other types of leukemia (87, 135). Nevertheless, Walker et al. found that DNMT3A is one of the sixty-three identified mutated driver genes in early MM development (136). In line with this study, a very recent study also reported the presence of a pathogenic DNMT3A mutation in the MGUS stage (137). Furthermore, the mutation frequency in DNMT3A was shown to increase upon relapse (87). On the transcriptional level, two studies demonstrated DNMT3A downregulation in MM and PCL, correlating with poor OS (**Table 2**) (79, 81). In contrast, Amodio et al. reported increased DNMT3A levels in MM and PCL patients compared to healthy controls. In addition, they provided evidence that miR-29b is a direct regulator of DNMT3A and that targeting DNMT3A using miR-29b mimics reduces MM cell growth (138). In line with the study of Amodio et al, Walker et al. also showed upregulation of DNMT3A in MM. This DNMT3A upregulation was particularly observed in the t(4,14) subgroup of patients and was the result of hypomethylation (80). Furthermore, the recent study from Luzna et al. also showed increased DNMT3A levels in MM patients compared to healthy controls and a trend to increased DNMT3A levels in ND and relapsed MM patients (the active phase of MM) compared to those in remission, thus showing its potential as a biomarker of MM progression (**Table 2**) (88). However, so far, the exact role of DNMT3A in MM biology has not yet been identified.

##### • DNMT3B

An oncogenic role for DNMT3B is observed in several hematological cancers, including T-ALL, AML, and Burkitt and diffuse large B-cell lymphoma (DLBCL), where increased DNMT3B levels are reported to be mostly the result of increased MYC-levels. Moreover, in AML, DNMT3A/B was also shown to be a direct target of miR-29b and forced miR-29b overexpression resulted in a decrease in DNMT3B and global methylation levels, resulting in the re-expression of some tumor suppressor genes including p15 (139). In T-ALL, silencing DNMT3B using DNMT3B shRNA reduced cell viability and cell growth, as evidenced by a decrease in the number of cells in the S-phase and an increase in the levels of CDKN1A (p21CIP1), CDKN2B (p15INK4b), CDKN2A (p16INK4a), and CDKN2D (p19INK4d) (140). Moreover, both in AML and DLBCL, high DNMT3B levels are correlated with a bad prognosis and a more aggressive disease (141, 142). However, while most studies indicate that DNMT3B has oncogenic properties in hematological cancers, some studies have also reported the opposite (143–145). For example, Dnmt3b deletion in the MLL-AF9 driven AML mouse model led to accelerated progression (146). Furthermore, Dnmt3b haploinsufficiency in mice resulted in the development of various hematologic malignancies, including TCL (145). In MM, Amodio et al. reported an inverse correlation between miR-29b and DNMT3B levels and showed that targeting DNMT3A/B using miR-29b mimics reduces MM cell growth, indicating that DNMT3A/B has an oncogenic role in MM (138). Furthermore, the enhanced stemness of MM cells observed upon coculturing them with granulocytic myeloid-derived suppressor cells (G-MDSC), was recently suggested to be the result of increased piRNA-823 and DNMT3B levels (147). However, to the best of our knowledge, the exact role of DNMT3B in MM biology and disease progression has not yet been thoroughly investigated.

#### - Erasers:

##### • TET2

TET2 is by far the most investigated TET family member, as it is the one found most frequently mutated. TET2 mutations can either be heterozygous or homozygous and appear to be very heterogeneous, encompassing nonsense and missense mutations, frame shift mutations, and in-frame deletions. Most of these mutations are loss-of-function mutations, leading to a reduced dioxygenase activity and thus a significant decrease in global 5hmC levels (148). These inactivating TET2 mutations suggest a tumor suppressive role for the TET2 protein, which is confirmed by the spontaneous development of myeloid, T-cell, and B-cell malignancies in a TET2 KO mouse model (149). TET2 mutations are frequently observed in hematological cancers, like myeloproliferative neoplasms (MPNs), chronic myelomonocytic leukemia (CMML), DLBCL, and AML (150–152). In contrast, in MM, TET2 mutations were only observed in about 1% of the patients from the Myeloma XI clinical trial (MyXI) (87). Nevertheless, Walker et al. identified TET2 as one of the sixty-three identified mutated driver genes, indicating that TET2 mutations are also an early event in MM development (136). Furthermore, the mutation frequency in TET2 was shown to increase upon relapse (115). Importantly, the prognostic value of the TET2 mutations remains controversial. In MDS, one study showed a correlation between TET2 mutations and a more favorable prognosis, while other studies showed no impact of TET2 mutations on the prognosis in MDS and AML (153–155) or even an inverse correlation (156). For MM, no prognostic potential was shown for TET2 mutations on its own, but Pawlyn et al. found that mutations in any of the DNA methylation modifiers (TET1/2/3, IDH1/2 or DNMT1/3A/B) correlated with a shorter OS (87). Of note, TET2 mutations are often found in combination with several other mutations, like NPM1, FLT3-ITD, FLT3-TKD, RUNX1, CEBPA, CBL, and KRAS (156). The differential combination of TET2 mutations with these other mutations might be a possible explanation as to why outcomes differ and why the prognostic potential remains controversial. Apart from the loss-of-function mutations, aberrant expression levels of TET2, although less frequent, have also been observed in hematological cancers. In MDS, downregulation of TET2, irrespective of the presence or absence of TET2 mutations, has been reported, while in AML patients, TET2 expression levels are significantly increased (157, 158). For MM, an increase in TET2 levels correlating with a better OS has also been observed (**Table 2**) (89).

##### • TET1 & TET3

Although mutations in TET1 and TET3 have been observed in some hematological malignancies (like AML, T-ALL, and chronic lymphocytic leukemia (CLL) for TET1 and MDS, myeloproliferative neoplasms, CMML, and B-cell acute lymphoblastic leukemia (B-ALL) for TET3) and were reported to be enriched in relapsed B-ALL cases, TET1 and TET3 mutations are in general quite infrequent (150, 159, 160). In contrast, TET1 and TET3 aberrant expression are more frequently observed in hematological malignancies. For TET1, overexpression has been reported in both MLL-rearranged leukemia and cytogenetically normal AML patients and was found to be correlated with a poor OS in the latter group, thus suggesting a pro-oncogenic role for TET1 in hematological cancers (161, 162). In contrast, Zhang et al. showed that TET1 expression is significantly reduced in AML patients (158). Moreover, hypermethylation and thus transcriptional silencing of TET1 is also observed in B-cell lymphoma, where its tumor suppressor activity was demonstrated *in vivo*, as KO of TET1 resulted in decreased survival of the mice (163). For TET3, a downregulation in TET3 levels was observed in CLL, but no significant correlation with OS was found although TET3 low expressers tended to have a worse OS (164). In contrast, TET3 overexpression was observed in AML and was associated with a longer disease-free and OS compared to patients with low TET3 expression (158). Furthermore, TET3 levels positively correlated with CDKN2B, ZIC2, and miR-196a levels, which appear to have anti-leukemic effects, thus suggesting a tumor suppressive role for TET3 in AML (158). However, more recently, TET3 overexpression in AML cell lines was shown to promote AML growth, suggesting rather an oncogenic role for TET3 (165). Of interest, a recent study in MDS showed an inverse correlation between TET2 and TET3. The authors suggest that the TET3 levels increase as a kind of compensation mechanism for the loss of TET2 expression and a lack in TET3 compensation correlated with high-risk features (increase in percentage of bone marrow blasts) and a poor outcome (157). In contrast, a recent study in AML patients showed a positive correlation between TET2 and TET3 and KO of both TET2 and TET3 in hematopoietic precursor cells in mice resulted in an almost complete loss of 5hmC and the emergence of myeloid leukemia (158, 166). Thus, it appears that the role of both TET1 and TET3 in hematological cancers (either oncogenic or tumor suppressive) is disease/context dependent and remains to be identified for MM.

## Involvement of DNA methylation modifiers in normal plasma cell differentiation, MM cell plasticity, and MM stemness

It is well-known that the epigenetic machinery tightly regulates the differentiation and maturation of hematopoietic stem cells (HSC) to mature B-cells and PCs. Dysregulation of the epigenetic machinery during this normal PC differentiation process is therefore linked to various B-cell related disorders. In MM, the tumor population is composed out of different subpopulations that differ in maturation stage, clonogenic capacity, and drug sensitivity (29, 167, 168). Importantly, it is believed that there exists a certain degree of ‘epigenetic plasticity’ between these different subclones, allowing the reprogramming/dedifferentiation of the terminally differentiated MM cells into the more immature and resilient subclones and vice versa upon treatment pressure (167, 169). Below we will briefly discuss the reported role of the DNA methylation modifiers in normal PC differentiation and MM cell plasticity and stemness.

### Role of the DNA methylation modifiers in normal plasma cell differentiation

The HSC present in the BM give rise to pro-B-cells and mature B-cells, which will then, upon antigen encounter further differentiate and mature into memory B-cells, expressing CD19 but not CD38, and plasmablasts, expressing both CD19 and CD38. The plasmablasts will then re-enter the BM to undergo terminal differentiation towards mature, non-dividing and immunoglobulin-secreting PCs. These long-living mature PCs are CD19-/CD38+/CD138+/Xbp1s+ (170). During the normal PC differentiation process, many epigenetic changes will take place. A prerequisite for the terminal differentiation of mature B-cells into fully mature PCs, is the shift from a B-cell transcriptional program, that maintains the B-cell phenotype (PAX5 and BCL-6), towards a PC specific transcriptional program (IRF4, Blimp-1, and Xbp1); with Blimp-1 as the master TF in PC generation (31, 171, 172). Blimp-1 shuts down the B-cell expression program by silencing over more than 250 B-cell specific genes, including PAX5 and BCL-6, by recruiting various co-repressors of the epigenetic machinery. Moreover, a recent study also indicated the importance of TET2/3 in PC differentiation, through demethylation of the IRF4 locus resulting in high IRF4 levels (173). In addition, DNMT3A/B also proved to play an important role in the repression of the B-cell expression program that is necessary for the B-cell activation and PC differentiation. DNMT3A/B deficient B-cells showed significantly less DNA methylation upon PC maturation compared to their normal counterparts due to failure of *de novo* DNA methylation. This lack of *de novo* DNA methylation resulted in increased chromatin accessibility at both B-cell and PC factors, including respectively PU.1, and IRF4 and E2A (174). In addition, changes in the expression levels of several of the DNA methylation modifiers have been observed during normal B-cell and PC differentiation. For example, DNMT1 and DNMT3B levels are upregulated and DNMT3A levels are downregulated during the transition from the naive to the germinal center (GC) B-cell stage and return to normal again in the post-GC memory B-cell. During the transition from memory B-cells to PCs, the DNMT3A levels will then decrease again, while the DNMT3B levels are increasing, although to a lesser extent than in the transition from the naive to the GC B-cell stage (175). Furthermore, using RNA sequencing analysis, upregulation of DNMT3B, TET1, IDH1/2, MBD1, and ZBTB38 during the transition of memory B-cells into PCs has been documented (176). In line with the changes in the expression levels of the DNA methylation modifiers, a global shift towards hypomethylation is observed during B-cell differentiation and this especially at the later stages, including the memory B-cell and the PC stage (177, 178). However, although the PC and the memory B-cells have a similar methylome, they appear to have a very different transcriptional program (175). This indicates that, as mentioned above, other epigenetic modifiers are also at play in the differentiation of memory B-cells to fully mature PCs. Indeed, several histone deacetylases (HDAC) and histone methyl transferases (HMTs), such as G9a, Enhancer of Zeste Homolog 2 (EZH2) and LSD1, have been shown to be involved in normal PC differentiation as well (176, 179).

### Role of the DNA methylation modifiers in MM cell plasticity and MM stemness

As mentioned earlier, the MM cell population is composed out of different subpopulations with each subpopulation differing in the level of maturation, response to treatment (drug sensitivity), transcriptional profiles, and clonogenic potential. In general, four co-existing MM subpopulations have been suggested, namely plasmablasts, which are CD19+; pre-PC, which are CD19- and CD138-; CD138low PCs; and the more mature CD19-/CD138+ PCs, which make up the bulk of the MM cells (170). Importantly, Chaidos et al. proposed a bidirectional transition between the pre-PCs and the mature PCs *in vivo* in NOD.Cg-Prkdc^scid^ Il2rg^tm1Wjl^/SzJ (NSG) mice, with the pre-PC showing an enrichment in genes encoding for epiplayers, such as histone acetyltransferases (HATs), HDACs, HMTs, histone demethylases (HDMs), and the methylation reader CCCTC-binding factor (CTCF) compared to the PC. These findings are suggestive of an epigenetic plasticity controlling the reversible and bidirectional transition between the more mature MM cells and the less mature myeloma cells, resulting in a PC/pre-PC equilibrium. Importantly, the authors also showed that the pre-PC are significantly less sensitive to chemotherapy combined with the PI Bz than the PC, suggesting that the mechanisms involved in this epigenetic plasticity are playing an important role in developing DR against the current MM therapies (167).

Moreover, it is strongly believed that the subpopulations of less mature MM cells have stem-like properties and are responsible for MM tumor initiation and propagation. Hence, these less mature subpopulations are thought to comprise the so-called MM stem cells (169, 180). Cancer stem cells (CSC) are well-known for their quiescence, self-renewal, and drug-resistant capacity, contributing to tumor aggressiveness, treatment resistance, and tumor recurrence (181). However, the exact nature of this MM stem cell population, including the surface markers, is still largely debatable (182). Increasing evidence is indicating that dysregulation of the epigenetic machinery, including aberrant expression and/or mutations in DNA methylation modifiers, is playing a role in CSC maintenance in both solid and hematological cancers (183). In MM, the enhancer regions of B-cell specific genes such as PAX5 and BATF are hypermethylated in MM cells compared to their normal counterparts. Importantly, these enhancers are also highly methylated in stem cells. This suggests that the MM cells (or at least a subfraction of them) either retain or regain stem cell features *via* epigenetic mechanisms. In line, a recent study suggested a role for DNMT3B in MM cell stemness. In short, granulocytic-myeloid-derived suppressor cells (G-MDSCs) cocultured with MM cells were shown to increase DNMT3B expression through piRNA-823 in the myeloma cells and increase their tumorigenic potential. Silencing of piRNA-823 led to decreased DNMT3B levels and a decreased stemness potential. The later was also supported by the decreased levels of CSC related genes, including NANOG, OCT4, and SOX2 (147). Moreover, both in MM cell lines and primary MM cells, an increase in clonogenic potential was observed for the residual cells following pomalidomide treatment. Further analysis revealed increased SOX2 and decreased levels of the methylation reader MBD3 in this population of residual myeloma cells, together with a clear deregulation of embryonal stem cell pathways. In line, MBD3 silencing by siRNA resulted in an increase in the clonogenic potential in myeloma cell lines (184). Finally, treatment of MM cells with berberine, a naturally occurring isoquinoline alkaloid, was recently shown to reduce the clonogenic potential of human myeloma cell lines (HMCLs) by inducing degradation of the DNA methylation reader UHRF1. In line, silencing UHRF1 in HMCLs using siRNA was also able to reduce their colony formation ability (185).

## DNA methyltransferase inhibitors

Since epigenetic modifications are reversible, they represent interesting targets to (partially) reprogram the MM cells back to their normal counterparts. Over the past two decades, several epigenetic modulating agents (EMAs) have been developed and investigated for their anti-myeloma activity. Below, we summarize the current and upcoming epigenetic therapies, focussing on the DNA methyltransferase inhibitors (DNMTi), alone or in combination with SoC agents and/or novel promising agents. Two main classes of DNMTi are available, namely the nucleoside analogs and the non-nucleoside analogs.

### DNMTi as single agents

#### The nucleoside analogs

By far the most clinical advanced DNMTi are the nucleoside analogs azacytidine (AZA) and decitabine (DAC). At the moment, AZA and DAC are already approved by the Food and Drug Administration for the treatment of MDS and other leukemias such as AML (186). Both AZA and DAC are incorporated into the genome upon DNA replication. In general, AZA and DAC have two modes of actions. First, upon treatment with relatively low concentrations, the DNMTs are trapped by the incorporated cytosine analogs, leading to their degradation. This process results in the depletion of DNMTs in the nucleus, leading to genome wide DNA hypomethylation and re-expression of tumor suppressor genes, which in turn reduces tumor growth and survival (187). Second, upon treatment with high AZA and DAC concentrations, massive protein-DNA cross-links are formed, leading to DNA replication fork stalling and the induction of a DNA damage response followed by cell death. In MM, AZA treatment led to p16 re-expression, a G0/G1 phase arrest and caspase-mediated apoptosis. In addition, AZA also suppressed the IL6 and NFkB signaling pathways (**Table 4**) (188). On the other hand, DAC treatment led to the re-expression of p15, p27, and p21 and the phosphorylation of p38 MAP kinase, which consequently led to both a G0/G1 and a G2/M phase arrest (11, 189, 190). We showed that, at high concentrations, DAC also induces DNA damage in MM cell lines. However, some of the cell lines were able to partially repair the DNA lesions because of their increased homologous recombination (HR) and/or non-homologous end joining (NHEJ) activity (as evidenced by the increased RAD51 and 53BP1 foci formation upon DAC treatment), thus resulting in attenuated cytotoxicity (**Table 4**) (190). In addition, we also demonstrated potent *in vivo* anti-MM activity in the 5T33MM mouse model, as evidenced by the significant higher survival rates in the DAC treated mice (190). Finally, a recent study also showed that DAC targets the monocytic myeloid derived suppressor cells (M-MDSC) and this both *in vitro* and *in vivo*. In line, combination of DAC treatment with MDSC targeting resulted in a stronger decrease in *in vivo* tumor growth compared to both single agents (202). However, despite these promising preclinical results, clinical trials so far showed a lack of efficacy when AZA or DAC were used as monotherapy in relapsed MM patients (**Table 5**). This can be partially explained by the fact that AZA and DAC are both quite unstable in aqueous solutions. Therefore, attempts were made to improve the efficacy of these two compounds (203).

**Table 4:** Overview of the DNMTi and TETi in preclinical development in MM.

	Anti-MM effect	References
DNMTi		
**The nucleoside analogs**		
AZA	p16 re-expression, G0/G1 phase arrest, caspase-mediated apoptosis and suppression of IL6 and NFkB signaling pathways	(188)
DAC	Re-expression of p15, p27, and p21 and phosphorylation of p38 MAP kinase, G0/G1 and G2/M phase arrest and induction of DNA damage	(11, 189, 190)
guadecitabine	Increased expression of miR-375 which is well-known to target PDPK1 No functional outcome reported	(191)
zebularine	Reduction of DNMT3A & DNMT3B levels resulting in reduced DNA methylation levels and reduced cell viability	(192)
CP4200	N.A	(65, 193)
5,6dihydroazacytidine	N.A.	(65)
5F-CdR	N.A	(65)
**The non-nucleoside analogs**		
mithramycin A	G0/G1 phase arrest and anti-angiogenic effects	(194)
Nanaomycin A	DNMT3B specific inhibitor Reduces global DNA methylation levels, induces cytotoxicity and reduces the number of MM CSC	(195)
EGCG	Inhibits EZH2 and decreased proliferation and increased apoptosis, effect on DNMT enzymes in MM unknown	(196–198)
RG108	N.A	(65)
procaine	N.A.	(65)
SGI-1027	N.A	(65)
NSC 14778	N.A.	(65, 117)
NSC 106084	N.A	(65, 199)
**TETi**		
C35 compound	N.A.	(200)
TETi76	N.A.	(201)

DNMTi, DNA methyltransferase inhibitor; TETi, Ten-eleven translocation inhibitor; AZA, azacytidine, DAC, decitabine; 5F-CdR, 5-Fluoro-2’-Deoxycytidine; EGCG, (–)-epigallocatechin-3-gallate; CSC, cancer stem cells; N.A., not applicable.

**Table 5:** Completed/terminated and ongoing clinical trials in MM testing DNMTi.

	NCT/ACTRN number	Treatment	Clinical Trial	Disease	Number of enrolled patients	Start-end date	Status	Outcome
**Completed/terminated studies**								
**Single treatment**								
	NCT00412919	AZA	Phase II	R/R MM	7	2006-2008	Terminated	Little to no effect but early termination limits the conclusions
	NCT00545519	AZA	Phase I	R/R MM	6	2006-2008	Completed	N.A.
	NCT00652626	AZA	Phase I	MDS, AML, MM, NHL, HL, and solid tumors	31	2008-2012	Completed	N.A.
	NCT00761722	AZA	Phase I	MDS, CMML, AML, Lymphoma and MM	31	2008-2016	Completed	Oral AZA administration is safe and bioavailability and other PK parameters are not meaningfully affected by food
	NCT01908387	AZA	Phase I	MDS, CMML, AML, MM, NHL, and HL	2	2013-2015	Terminated	Early termination due to slow accrual of the patients
	NCT02223052	AZA	Phase I	Solid and hematological malignancies including MM	89	2014-2018	Completed	Bioavailability and other PK parameters are not meaningfully affected by food
	NCT00002980	DAC	Phase I	Melanoma, MM, MDS, leukemia, lymphoma, CMD	N.A.	1997-N.A.	Completed	N.A.
	NCT00942422	EGCG	Phase II	MGUS/SMM	8	2009-2012	Terminated	Early termination due to slow accrual of the patients
**Combination treatment**								
	NCT01050790	AZA + Len followed by ASCT	Phase II	R/R MM	17	2010-2016	Completed	6 out of 11 patients showed CTA upregulation in BM or CD138+ cells and all three patients tested showed a CTA-specific T cell response that persisted following ASCT Epigenetic induction of an adaptive immune response to CTA is safe and feasible
	NCT01155583	AZA + Len + Dex	Phase I/II	R/R MM	45	2010-2018	Completed	ORR of around 23%, but with the cost of grade 3/4 toxicities in 58% of the patients
	ACTRN12613000283774	AZA + Len + Dex	Phase Ib	R/R MM with history of Len failure	24	2013-2016	Terminated	Patient recruitment difficulties ORR of 37.5%, no significant toxicities
**Ongoing studies**								
	NCT04174196	AZA + Len + Radiation	Phase II	Plasmacytoma (of bone), MM	20*	2019-2023	Recruiting	Not yet available
	NCT04407442	AZA + Dara + Dex	Phase II	R/R MM	23*	2020-2023	Recruiting	Not yet available
	NCT05065866	AZA + Duvelisib	Phase I	NHL, MM, Hodgkin Lymphoma, Lymphocytic Leukemia	30*	2021-2024	Recruiting	Not yet available

MGUS, monoclonal gammopathy of undetermined significance; SMM, smoldering multiple myeloma; R/R MM, relapsed or refractory multiple myeloma; MDS, myelodysplastic syndromes; AML, acute myeloid leukemia; NHL, non-Hodgkin lymphoma; HL, Hodgkin lymphoma; CMML, chronic myelomonocytic leukemia; CMD, chronic myeloproliferative disorders; AZA, azacytidine; DAC, decitabine; EGCG, (–)-epigallocatechin-3-gallate; Len, lenalidomide; Dex, dexamethasone; Dara, daratumumab; ASCT, autologous stem cell transplantation; BM, bone marrow; PK, pharmacokinetic; ORR, overall response rate; CTA, cancer testis antigens; N.A., not available. * Estimated number of patients that will be enrolled.

A more stable version of DAC is a dinucleotide of DAC called guadecitabine (SGI-110). Guadecitabine is resistant to cytidine deaminase and has been tested in a phase 2 clinical trial of 107 newly diagnosed AML patients and a phase 2 clinical trial of fifty-five adult relapsed/refractory AML and MDS patients. The first study showed a complete response rate of more than 50%, while the second study showed responses in 14.3% of the patients resulting in prolonged OS. Other phase 1 and 2 clinical trials that are using guadecitabine in AML are currently ongoing (143). In MM, treatment of human cell lines with guadecitabine resulted in increased expression of miR-375, which is well-known to target 3-phosphoinositide-dependent protein kinase 1 (PDPK1). In line, guadecitabine also reduced PDPK1 levels (**Table 4**) (191). However, the authors did not report about the functional outcome. A second, more stable nucleoside analog that has been evaluated in MM is zebularine, which appears to be less toxic compared to AZA and DAC (204). In MM, zebularine reduced both DNMT3A and DNMT3B levels, resulting in reduced DNA methylation levels and reduced MM cell viability (**Table 4**) (192). However, so far, these improved versions of the nucleoside analogs have only been tested *in vitro* in MM and part of the reason for this might be the limited bioavailability observed in different species (205). Other more stable DNMTi such as CP4200, 5,6dihydroazacytidine, and 5-Fluoro-2’-Deoxycytidine (5F-CdR) have also been described, but have so far not been tested yet in MM (**Table 4**) (65, 193).

#### The non-nucleoside analogs

A second major drawback of all the nucleoside analogs is that cell cycle progression is needed for the incorporation of these cytosine analogs into the DNA. However, myeloma cells are well-known for their relatively low proliferation rates, thus implying that only a minor fraction of the myeloma cells will be affected at a given time (190, 206). This shows a need for DNMTi that are not dependent on incorporation into the DNA for their activity. Currently, a small number of DNMTi have been described that block the catalytic activity of the DNMTs by directly binding the DNMTs or, in some cases, by binding CpG rich sequences. Apart from the fact that these so-called non-nucleoside DNMTi do not require incorporation in the DNA to exert their catalytic activity, another major advantage of these non-nucleoside analogs is that, since no formation of DNA-DNMT adducts leading to DNA damage takes place, lower levels of cytotoxicity are expected, thus resulting in a larger therapeutic window compared to the nucleoside analogs. Below, we will zoom in on the most interesting non-nucleoside analogs that have been currently tested in MM.

A first non-nucleoside DNMTi that was tested in MM is mithramycin A. Treatment of MM cell lines with mithramycin A resulted in an arrest of the cells at the G1/S transition point. Moreover, mithramycin A was shown to exert anti-angiogenic effects both *in vitro*, using the endothelial cell migration assay and the rat aortic ring assay, and *in vivo*, using the 5TGM1 MM mouse model (**Table 4**) (194). However, it is still under debate whether mithramycin A reversibly binds CpG-rich DNA sequences or whether it binds the catalytic domain of DNMT1, leading to its depletion. Another novel DNA hypomethylating drug that falls under the class of non-nucleoside analogs is Nanaomycin A. Kuck et al. showed that this antibiotic of the anthracycline group is a DNMT3B specific inhibitor that reduces global DNA methylation levels and induces cytotoxicity in human cancer cell lines (**Table 4**) (195). In addition, as mentioned above, a more recent study showed that Nanaomycin A significantly reduces the amount of MM CSC, thus suggesting that inhibition of DNMT3B using Nanaomycin A would be effective in targeting the CSC in MM (147). Another non-nucleoside analog is (–)-epigallocatechin-3-gallate (EGCG). This compound is well-known to have anti-MM effects as evidenced by decreased proliferation and increased apoptosis of MM cells upon EGCG treatment (196, 197). EGCG has already been tested in a phase 2 clinical trial in MGUS and SMM patients, but was terminated early due to low patient enrolment (**Table 5**). Although EGCG has been shown to inhibit the HMT EZH2 in MM, so far, no study has yet investigated the effect of EGCG treatment on the activity of the DNMT enzymes in MM (**Table 4**) (196, 198). Other non-nucleoside inhibitors that might be interesting to test for the treatment of hematological cancers, including MM, are RG108, procaine, SGI-1027, NSC 14778, and NSC 106084 (**Table 4**) (65, 199). For a detailed description of these non-nucleoside analogs we refer to the review written by Foulks et al. (65).

### DNMTi in combination therapy

Given the presumed role for epigenetic modifications in MM cell plasticity, epigenetic alterations have also been suggested to play a role in developing DR against current MM therapies. This provides the rationale for combining one or more epigenetic modulating agents (EMA) with SoC agents to overcome or even prevent relapse. Here, we will focus on the combination of DNMTi with SoC agents, other EMAs and/or new (immuno)therapies that have already been tested in MM.

As mentioned earlier, we previously showed that the increased HR and NHEJ activity in MM cells partially protects the MM cells from DAC-mediated cytotoxicity. However, we also showed that the histone deacetylase inhibitor (HDACi) JNJ-585 (also known as quisinostat) strongly enhances the *in vitro* and *in vivo* anti-MM activity of DAC by decreasing HR DNA repair (190, 207). Moreover, using gene expression profiling of DNMTi and/or HDACi treated MM cells, we later on constructed a gene-expression based score to predict patient outcome and MM sensitivity toward HDACi/DNMTi combination treatment. Patients with a low combo score were characterized by a mature BMPC gene signature, whereas patients with a high combo score were characterized by a proliferation and MYC-associated gene signature and a worse OS (high-risk patients) (**Table 2**) (84). Nevertheless, the MM cells from this high-risk group showed a higher sensitivity towards the combination of the HDACi quisinostat or trichostatin A (TSA) and the DNMTi DAC. Mechanistically, we showed that the HDACi/DNMTi combination resulted in the reprogramming of the MM cells by strongly downregulating IRF4 and MYC and inducing a normal BMPC gene expression profile. Importantly, this strong conjoined downregulation of MYC and IRF4 expression was only observed after the combination treatment and not after DNMTi or HDACi treatment alone (208). Together, these findings provide a strong rationale for the targeting of MM cells with at least two different EMA classes. In line, a more recent study designed epigenetic compounds simultaneously targeting HDACs and DNMT1 (compound 12a) on the one hand and HDACs, DNMT1, and the methyltransferase G9a (compound 9a) on the other hand. Both the dual and triple epigenetic inhibitor resulted in reduced proliferation of MM cells. Furthermore, both inhibitors led to an increase in H3K9ac levels and to increased hypomethylation, whereas compound 9a also led to a significant reduction in the levels of the H3K9me2 mark. Compound 12a was also found to significantly reduce tumor growth in the MM1.S xenograft mouse model. Unfortunately, compound 9a could not be tested *in vivo* as 9a proved to be lethal for the mice (209).

As IMiDs are well-known to target IRF4 and MYC, our findings also suggest that the combination of IMiDs with DNMTi/HDACi combo treatment could be of therapeutic interest for high-risk MM patients. In line, Dimopoulos et al. recently showed that dual inhibition of DNMTs and the HMT EZH2 using AZA and EPZ-6438 respectively overcomes both intrinsic and acquired IMiD resistance and this independently of cereblon (CRBN). Importantly, the IMiD resistant MM cells were found characterized by increased genome-wide DNA methylation levels and reduced chromatin accessibility and thus reduced gene expression levels. Combination of AZA with EPZ-6438 re-sensitized the IMiD resistant MM cells to both lenalidomide and pomalidomide by reversing the reduced chromatin accessibility (210). A recent study also found that hypermethylation of an active intronic CRBN enhancer was more pronounced in IMiD-refractory MM. Treatment of two MM cell lines with DNMTi resulted in the demethylation of this CRBN enhancer region, thus resulting in increased sensitivity against lenalidomide (211). Together, these results provide evidence that IMiD-acquired resistance in MM is, next to genetic mutations in CRBN, also driven by epigenetic mechanisms and that one or more EMAs together with IMiDs can restore sensitivity.

Apart from (re)boosting IMiD activity, DNMTi have also been shown to potentiate the activity of other SOC and novel agents. A pre-clinical study using the MM cell line RPMI 8226 showed that the DNMTi DAC also enhances the anti-myeloma activity of Bz, as evidenced by a stronger reduction in proliferation and stronger increase in apoptosis compared to both single agents (212). Moreover, a more recent study showed that the combinatory effect observed upon combining DAC and Bz is regulated, at least in part, by the Wnt/β-catenin signaling pathway, as re-expression of the Wnt antagonists SFRP3 and DKK1 was observed upon DAC treatment (213). In addition, DAC has also been shown to restore drug sensitivity in the dexamethasone resistant OPM1 cell line, by inducing re-expression of the tumor suppressor gene RASD1 (115). Furthermore, both AZA and DAC were only very recently shown to restore sensitivity to the monoclonal anti-CD38 Ab daratumumab in MM cell lines, by reverting epigenetic silencing of CD38 upon daratumumab treatment (214). Finally, DAC treatment was recently also shown to sensitize myeloma cells to CAR T therapy. DAC treatment resulted in global hypomethylation and increased expression of the cancer testis antigen NY-ESO-1, thereby enhancing cell lysis upon DAC and NY-ESO-1-specific CAR combination treatment (215). These recent results suggest that (pre-)treatment with DAC might sensitize the myeloma cells to daratumumab and CAR T-cell therapy.

So far, clinical trials have mainly investigated the efficacy of AZA in combination with lenalidomide and/or dexamethasone (**Table 5**). Reu et al. found that low-dose AZA (subcutaneously) in combination with lenalidomide and/or dexamethasone yielded response rates of about 23% in patients with relapsed or refractory MM, but with the cost of grade 3/4 toxicities in about half of the patients (216). In addition, another study investigating the combination of oral AZA together with lenalidomide and/or dexamethasone in relapsed or refractory MM patients with a history of lenalidomide failure showed overall response rates of about 37.5% without significant toxicities. The author suggested that these superior results, compared to the study from Reu et al, were the result of the extended exposure to AZA (217). Finally, a recent case report described the treatment of a MM patient with a combination of AZA and lenalidomide followed by a combination of AZA and daratumumab upon relapse to be successful (218). Furthermore, a clinical trial investigating a combination therapy combining AZA with e.g., daratumumab and dexamethasone is currently ongoing (**Table 5**). Clinical trials combining DNMTi with Bz or CAR T-cells in MM are still awaited.

## Conclusion & future perspectives

It has become apparent that epigenetic defects, including global DNA hypomethylation and locus specific DNA hypermethylation together with aberrant expression and/or mutations in the DNA methylation modifiers, play an important role in MM onset, progression, and relapse. Moreover, it is becoming increasingly apparent that aberrant DNA methylation patterns/modifiers also play a role in acquiring a heterogeneous MM cell population, encompassing both the mature MM cells and the more immature MM cells characterized by their drug-resistant capacity, and allows for the bidirectional transition between these two states. This epigenetic plasticity allows the MM cells to adapt to their environment, including treatment pressure, and thus escape from all currently available MM therapies. However, although the aberrant DNA methylation patterns and modifiers are clearly major obstacles for curing MM, they also represent great opportunities. Firstly, DNA methylation modifiers represent attractive targets to overcome or delay (acquired) MM cell DR. Indeed, (pre)clinical studies have repeatedly shown that combining DNMTi with either lenalidomide, dexamethasone, Bz, or HDACi is promising for (re)sensitizing the MM cells. Furthermore, DNMTi were recently also shown to upregulate the epigenetically silenced surface proteins used as targets in immunotherapies, including daratumumab and CAR T therapy. These findings advocate for the careful consideration of incorporating DNMTi in the current MM therapies. Moreover, it would also be interesting to find out whether DNMTi could also be of benefit to increase the efficacy of the new, emerging immunotherapies such as ADC and T-cell engagers. Secondly, it is well-known that DNA methylation patterns, as a consequence transcriptional patterns, change upon MM progression. Characterization of the DNA methylation patterns and/or methylation-regulated gene expression in MM patients might, on the one hand, predict response to treatment and OS and, on the other hand, identify those patients that might benefit most from DNMTi therapy. In fact, Moreaux et al. and we developed gene expression-based scores that predict not only patient outcome, but also primary MM cell sensitivity to HDACi and/or DNMTi treatment (84–86). Furthermore, a DNA methylation inference framework called MethSig was recently developed that was demonstrated to be superior in distinguishing stochastic DNA methylation changes, with no biological consequence, from likely driver DNA methylation changes in CLL and MM. When developing a prediction score based on the identified candidate DNA methylation drivers, a higher DNA methylation driver risk score was found associated with an adverse outcome in CLL patients (219). Currently, this DNA methylation driver risk score has not yet been constructed for MM. Hence, it will be interesting to further apply this MethSig tool on MM DNA methylation sequencing cohorts to further identify the oncogenic DNA methylation drivers in MM and to construct and test a MM DNA methylation driver risk score.

However, in order to seize these opportunities, some challenges must be tackled first. Since only a tip of the iceberg concerning the role of the different DNA methylation modifiers in MM disease progression and especially in relapse has thus far been revealed, it is of utmost importance to further investigate the role of these DNA methylation modifiers. In this regard, it will be important to, apart from the DNA methylation writers and erasers, not lose sight of the DNA methylation readers; a group of modifiers that has until now completely been neglected in MM. Recent evidence however points out that DNA methylation readers including readers from the methyl binding domain (MBD) family (MBD3), the SET and RING finger associated (SRA) domain protein family (UHRF1), and the zinc finger family (EGR1 and KLF4) are also involved in cancer pathogenesis. Moreover, in order to make it even more complicated, it is well-known that many DNA methylation modifiers have multiple splice variants/isoforms, with apparently each of these isoforms having distinct activities. At the moment, the role of these different isoforms and their consequences in MM disease is not yet explored. As these isoforms can regulate the activity of the default DNA methylation modifiers, it will be interesting to investigate the role of both catalytic active and inactive isoforms in MM disease. Furthermore, although preclinical (combination) studies showed promising results, clinical studies demonstrated that the pan-DNMTi are subject to a lack of efficacy and high toxicity profiles complicating the broad application of these agents in the clinic. Hence, more specific DNA hypomethylating drugs are urgently needed. Different novel DNMTi, encompassing both the newer nucleoside and non-nucleoside inhibitors, have already been developed and this number is only increasing. However, till today, only a small number of these inhibitors has been tested in MM. Moreover, when looking in the direction of the erasers, no TET inhibitors (TETi) have so far been tested in MM. Two promising TET inhibitors (TETi) have only very recently been identified, C35 compound and TETi76. Both compounds specifically target all three TET members (200, 201). Although one of the two compounds, namely TETi76, has already been tested in AML and was shown to restrict colony formation, neither compounds have yet been tested thoroughly in cancer. Further research is needed to provide insights into the applicability of TET inhibitors for the treatment of MM.

In conclusion, it is clear that the true potential of DNA methylation patterns and modifiers for MM patient stratification and therapeutic targeting is only starting to unfold itself. Although many challenges still need to be overcome, we envision that in time DNA methylation sequencing and the use of DNA methylation modulating agents will become common practice for the follow-up and treatment of MM.

## Author contributions

CM and EDB developed the design and arguments for the paper and drafted the manuscript. CM, LH, and EDB designed the figures. LH, AM, KV, EM, and KV revised the manuscript. All authors contributed to the article and approved the submitted version.

## Funding

This work was supported by Fonds voor Wetenschappelijk Onderzoek (FWO), Wetenschappelijk Fonds Willy Gepts (WFWG), and Strategic Research Programme (SRP48), K. De Veirman is a postdoctoral fellow of FWO (12I0921N).

## Conflict of interest

The authors declare that the research was conducted in the absence of any commercial or financial relationships that could be construed as a potential conflict of interest.

## Publisher’s note

All claims expressed in this article are solely those of the authors and do not necessarily represent those of their affiliated organizations, or those of the publisher, the editors and the reviewers. Any product that may be evaluated in this article, or claim that may be made by its manufacturer, is not guaranteed or endorsed by the publisher.
